# Virtual reality experiences, embodiment, videogames and their dimensions in neurorehabilitation

**DOI:** 10.1186/s12984-018-0461-0

**Published:** 2018-11-26

**Authors:** Daniel Perez-Marcos

**Affiliations:** MindMaze SA, Lausanne, Switzerland

**Keywords:** Virtual reality, Virtual reality experience, Virtual reality system, Videogames, Virtual embodiment, Neurorehabilitation

## Abstract

**Background:**

In the context of stroke rehabilitation, new training approaches mediated by virtual reality and videogames are usually discussed and evaluated together in reviews and meta-analyses. This represents a serious confounding factor that is leading to misleading, inconclusive outcomes in the interest of validating these new solutions.

**Main body:**

Extending existing definitions of virtual reality, in this paper I put forward the concept of virtual reality experience (VRE), generated by virtual reality systems (VRS; i.e. a group of variable technologies employed to create a VRE). Then, I review the main components composing a VRE, and how they may purposely affect the mind and body of participants in the context of neurorehabilitation. In turn, VRS are not anymore exclusive from VREs but are currently used in videogames and other human-computer interaction applications in different domains. Often, these other applications receive the name of virtual reality applications as they use VRS. However, they do not necessarily create a VRE. I put emphasis on exposing fundamental similarities and differences between VREs and videogames for neurorehabilitation. I also recommend describing and evaluating the specific features encompassing the intervention rather than evaluating virtual reality or videogames as a whole.

**Conclusion:**

This disambiguation between VREs, VRS and videogames should help reduce confusion in the field. This is important for databases searches when looking for specific studies or building metareviews that aim at evaluating the efficacy of technology-mediated interventions.

## Background

In the context of stroke rehabilitation, new training approaches mediated by virtual reality and videogames are usually discussed and evaluated together in reviews and meta-analyses for upper limb [1, 2], and balance and gait [3]. Certainly, the expected superiority of virtual reality over conventional therapy post stroke has been questioned when using off-the-shelf (e.g., Nintendo Wii) or ad-hoc videogames. This conclusion, however, is based on the wrong assumption that videogames deliver same experiences than virtual reality applications. In my opinion, this represents a serious confounding factor that may lead to misleading, inconclusive outcomes in the interest of validating these new solutions. Indeed, in Laver’s Cochrane article, a positive effect for virtual reality versus conventional therapy for improving upper limb function post stroke is found only when dedicated virtual reality based interventions, i.e. specifically designed for rehabilitation settings, are used. The effect vanishes when standard off-the-shelf videogames are considered. Indeed, the use of Nintendo Wii (but referring to it as virtual reality) often leads to a non-inferiority clinical outcome, being as effective as conventional therapy [4] or alternative playful interventions such as playing cards [5]. In another study with mobile-based and dedicated games (again referred to as virtual reality), partial functional and motor improvements were observed as compared to standard occupational therapy [6].

This heterogeneity in the reported virtual reality and videogames studies for neurorehabilitation calls for use of appropriate labelling for the approaches and variables assessed. A correct identification of the specific factors (and their weight) contributing to any eventual change post treatment are required for interpreting those changes and building further evidence on the specific solution. Therefore, in this paper I propose to reframe the traditional interpretation of the term virtual reality. I advocate disentangling two conceptual components that may help the field standardize its use: virtual reality experience (VRE) and virtual reality systems (VRS). I put emphasis on exposing fundamental similarities and differences between VREs and videogames, often mistakenly used as synonyms or exchangeable terms despite the different underlying interventional techniques and brain mechanisms they can enable. I then use neurorehabilitation as exemplary application field to discuss the implications of differentiating between them.

## Main text

### The evolving concept of virtual reality

Virtual Reality is likely today’s most powerful experiential technology available, i.e. a technology able to create immersive live experiences. Historically, virtual reality has been defined in terms of technological hardware, typically referring to computer-generated 3D realities implemented with stereoscopic displays and, sometimes, haptic gloves (see review in [7]). Soon, its definition evolved to include presence as the main characteristic and vividness measure of the experience [8].

As other forms of interaction with the virtual worlds were integrated into the setups, more generic definitions of virtual reality have been proposed. Weiss and colleagues wrote that “Virtual reality typically refers to the use of interactive simulations created with computer hardware and software to present users with opportunities to engage in environments that appear to be and feel similar to real-world objects and events” [9].

When used in applied neuroscience, definitions of virtual reality put additional focus on the multisensory stimulation. For instance, Henderson defined virtual reality as a “computer based, interactive, multisensory environment that occurs in real time” [10]. Here virtual reality enables the use of multisensory stimulations (visual, auditory, tactile, smell, kinesthetic, proprioceptive) to generate illusory realities, mostly related to the self (and body), in order to get the mind to believe (and accept) the virtual as real: “Virtual reality system components work in concert to create sensory illusions that produce a more or less believable simulation of reality” [11]. Slater has summarized these aspects to state that virtual reality is different from other forms of human–computer interface since the human “participates in the virtual world rather than uses it” [12].

Today, virtual reality has become a medium in itself, able to generate unique experiences. In turn, systems and technologies initially developed for virtual reality are nowadays being used in an increasing range of human-computer interaction forms in different domains (gaming, industry, training, healthcare, etc.). As the acceptance and use of virtual reality is spreading across disciplines, it is not surprising that the interpretation of the notion of virtual reality varies from one field to another, or even among different stakeholders within the same field. There is, thus, a danger of conceptual reductionism that creates wrong expectations that may lead to results misinterpretations of its use.

### Virtual reality experiences

A VRE is composed of a set of qualities that make the experience more or less real. Although the minimal requirements to experience a VRE are not fully defined yet, based on the literature I identify at least four groups of interrelated contributors: (i) immersion, (ii) interaction, (iii) sensorimotor contingencies and, as consequence of these, (iv) illusions.

#### Immersion

Immersion is most likely the first associated term to VREs. In virtual reality, nowadays the main contribution to technological immersion comes from how visual information is provided. Immersion, however, is not just a question of display type, size and field of view, and it can be enhanced by adding other sensory cues (e.g. auditory, tactile). Indeed, immersion is not specific to electronic displays. For instance, a book can provide a certain level of engagement by which readers mentally visualizing the described world or identifying with the protagonists’ fears or pain can experience certain level of immersion [13]. However, to elevate the reading experience to the VRE category, other elements like sensorimotor contingencies and illusions are required.

#### Interaction

The initial feelings generated after exposure to a new immersive environment (virtual or other) may equally vanish if this immersion is not fed by sensorimotor contingencies provided by natural and meaningful interaction with the environment. Natural interaction in VREs starts with visual exploration of the space. For instance, when using a head-mounted display (HMD) with integrated head tracker and turning the head around, participants can explore the virtual world as they would do in the real world. The level of interaction increases every time the system responds to the participants’ action, e.g. a virtual human looks back to the participant when she/he turns the gaze to the avatar. If a virtual representation of their body is included in the experience, when reaching to a virtual object, participants will expect the virtual arm to touch it and they will expect to feel its texture, shape and weight (e.g., with aid of a haptic data glove). When this tactile dimension is provided, the visuomotor interaction is enhanced with a new piece of congruent sensory information.

More elaborate ways of interaction may be provided by means of brain or other bodily inputs, mainly via brain-computer interaction and biofeedback, which enable participants to navigate through a virtual environment [14] or, for instance, to see their respiration reproduced by the chest movements of a virtual body in a synchronous fashion to enhance self-identification with the virtual body [15]. In another example of the use of visualization of interoceptive signals, Aspell and colleagues proposed synchronous cardio-visual stimulation, with a virtual body flashing in synchrony with participant’s heartbeats, to increase self-identification with the virtual body [16].

#### Sensorimotor contingencies

Sensorimotor contingencies refer to the actions that we carry out to perceive the world [17]. In VREs, they are often provided by our interaction with the environment, e.g. when leaning forward trying to see what there is hidden behind an object. Sensorimotor contingencies are directly involved in the generation of the sense of agency, i.e. the experience of being the author and initiator of our own actions [18]. This is particularly important for our body interaction with the world in VREs. For instance, our perception of the virtual world depends on the congruence between our actions and the sensory feedback resulting from them, e.g. we feel the stiffness of the virtual object that our virtual body representation is touching.

The spatio-temporal correlation of the concurrent multisensory stimuli represents a critical factor for the nervous system to interpret the environment [19]. Arbitrary spatio-temporal discrepancies created artificially in a VRE can also be used to facilitate activation of targeted brain networks to study specific pathological conditions or to potentially speed up the recovery process in rehabilitation settings (see [20] for a review).

#### Illusions

Illusion is defined as “an instance of a wrong or misinterpreted perception of a sensory experience” [21]. Within this context, VREs encompass a series of perceptual illusions related to the space, the environment and the self. The more senses congruently involved (i.e. stimulated) during the interaction, the higher the strength of the illusions generated.

Presence, also called Place Illusion, is the psychological product of technological immersion [11]. Presence is commonly defined as “the feeling of being there” [8, 17]. An ideal VRE would provide the ultimate level of immersion, creating an illusion of full physical presence in real or imagined worlds. More recently, Slater has further analyzed the original concept of presence, distinguishing between place illusion and plausibility illusion, i.e. the illusion that what is apparently happening is really happening to you [17]. This includes the self as an intrinsic element of the experience, as participants stop being mere spectators or controllers (if control over the virtual world and its objects is enabled) to become actors of the new reality. Indeed, immersion provides the boundaries within which place illusion or presence can occur and can be characterized by the sensorimotor contingencies that they support [17].

VREs can modify, add and substitute actual sensory information from reality for the experience to “feel real” [22]. VREs can even propose to replace the own body with a virtual representation. The illusion of having (i.e. feeling as if real) a body in a VRE has been tagged as virtual embodiment. The investigation of the mechanisms and the consequences (physical, cognitive, and even legal) of manipulating body perception through bodily illusions is the ultimate goal of a dedicated research field in cognitive neuroscience that started with the “rubber hand illusion” [23] and that rapidly expanded to virtual body illusions [24, 25]. For instance, manipulations of the bodily self can be used to examine the brain mechanisms that support body perception and integration in healthy subjects [26] and in the context of diverse pathologies, ranging from eating disorders [27] to neuropathic pain in paraplegia [28].

This sense of embodiment has been suggested to consist of three illusory components: the sense of self-location, the sense of agency, and the sense of body ownership [29]. Several conceptual models have been proposed to address how our nervous system decides to accept a fake (e.g., rubber or virtual) body as being its own. A combination of Bayesian models characterizing bottom-up and top-down neural processes seems to explain bodily illusions obtained via VREs [26, 30, 31]. While the illusion of presence can be easily generated by simply exposing the participant to an immersive virtual environment, the illusions related to the own body (our physical link to the outside world) require higher levels of sensorimotor contingencies.

Plausibility, place and virtual embodiment illusions together are probably the key differentiation elements of VREs versus other existing media and experiences. Although virtual embodiment (i.e., having a virtual representation of the own body) is not a sine qua non condition to elicit strong VREs, when a participant looks down when wearing an HMD, they expect to see their (virtual) body. Therefore, the occurrence of embodiment presupposes a certain level of presence, sensorimotor contingencies and plausibility illusion. Indeed, while high levels of presence can be achieved with none or low embodiment level, e.g. using third-person perspective, virtual embodiment provided through first-person perspective enables more accurate interactions in the virtual world [32].

#### VRE metrics

Strategies to measure the quality and strength of VREs and their components are currently shifting from subjective scales (usually questionnaires) to more objective behavioral and neurophysiological measures. For instance, event-related brain potentials have been suggested as a non-subjective measure of virtual embodiment [33]. In that study, brain activity in the motor cortex and readiness potential negativity were observed when an embodied virtual hand was threatened (but not the real hand), which was associated with the intention of the participant to move away the threatened (virtual) hand to avoid harm. Other examples of bodily responses used to measure the illusions generated in VREs include changes in electro-dermal activity (galvanic skin response), pupil dilation or skin temperature (see summary in [34]). However, all these measures mostly represent indirect measures of the assessed element, as illusions like presence or body illusions are subjective experiences (qualia) arising from multisensory stimulation, which cannot be directly measured [17].

### Virtual reality systems

VRS refers to a set of physical solutions or means to generate VREs. Historically, goggles or HMDs, motion capture and haptic gloves have been considered the identification signs for virtual reality. Gonzalez-Franco and Lanier recently suggested that the minimum instrumentation requirements to support illusory VREs should include continuously updated multisensory feedback (at least visual) with congruent sensorimotor contingencies to prevent violations of brain expectations [22]. Although stereoscopic 3D images displayed in highly immersive HMD are more effective in generating a strong and continuous VRE, wearing an HMD or 3D goggles does not guarantee having strong VREs unless the other components (interaction, sensorimotor contingencies, illusions) also take place. Conversely, VREs can also be experienced on flat screens despite their lower level of immersion.

Indeed, the recent new boom of virtual reality starting in 2012 is related to the mass-market arrival of two technologies initially developed for virtual reality applications: HMDs and, to a lesser extent, motion capture systems. HMDs can provide stereoscopic images for fully immersive experiences either isolating user’s vision from reality, which is occluded in the case of non-see-through HMDs, or displaying the surrounding world, e.g. using optical or video see-through HMDs for augmented and mixed reality. Motion capture represents a powerful tool to create visuomotor correlations between one’s own movements and those of an avatar. The gaming industry is particularly taking advantage of both technologies, in a field where interaction has been traditionally two-dimensional and non-natural (either with a simple mouse or with game controllers). In return, low-cost 3D motion tracking systems that were originally developed for gaming (Microsoft Kinect) and computer interaction (LeapMotion) are populating todays VRS, underlining an increasing confluence of these related fields.

By adding different VRS elements, the user can interact with the environment and its content (including persons) to increase the number and strength of sensorimotor contingencies. The more senses congruently involved (i.e. stimulated) in this interaction, the stronger the illusions generated. To integrate new interactions and sensorimotor contingencies into VREs, new tools are continuously widening the list of VRS components. Indeed, VRS have also borrowed advanced tools from other fields ranging from human- and brain-computer interaction, e.g. physiological signals for bio- and neurofeedback purposes, to neurostimulation techniques [35]. These signals can be used to measure different aspects of the VRE or to control exposure to adaptive virtual environments, e.g. for treatment of phobias or anxiety mitigation (see review in [36]). Slater and colleagues have recently published a guide with the technical and experimental building blocks required to generate powerful VREs, in particular embodiment illusions, ranging from motion capture to brain-computer interfaces [34].

### Embodied VREs for neurorehabilitation

VREs enclose many advantages for healthcare applications, in particular for neurorehabilitation. VREs can deliver sensorimotor training able to engage brain motor and perceptual areas as body-related visual feedback (alone or in combination with other multisensory stimulation, e.g. tactile and auditory) can be provided in real time. VREs using embodied feedback allow for manipulations of a virtual body that could have implications for neurorehabilitation [37]. Indeed, a critical ability of VREs is the possibility to promote changes in the perception of the own body by means of an illusion of ownership of a virtual body. For instance, illusions based on multisensory correlations can be extremely useful to apply VRE-based mirror visual feedback [38] and action-observation therapy for motor rehabilitation post stroke [39]. A recent study has shown that chronic stroke participants are more sensitive to embodiment illusions than healthy controls, i.e. they experienced stronger body ownership and sense of agency towards the fake hand in the rubber hand illusion [40]. This augmented sensitivity in stroke patients opens the space to further manipulations, which may not fully correspond with reality. Indeed, movement reproduction by the virtual body does not need to necessarily match participant’s ones. For instance, the visual amplification of the movement of the paretic limb seems to promote its use in chronic stroke patients [41], which also translated to changes in clinical outcomes [42].

In a preliminary study with 18 healthy subjects, fMRI data recorded during exercising with a VRE-based rehabilitation system providing embodied feedback has revealed activation in brain regions associated with motor control and consistent with those related to the human mirror neuron system [43]. Authors observed activations of the left supplementary motor area, the inferior parietal lobe and the inferior frontal gyrus, which have been shown to be active during execution, imitation, observation or motor imagery of goal-directed movements using action observation [44].

Concerning the evaluation of effectiveness of VRE-based interventions post stroke, one of the main issues is that often standard clinical scales are inappropriate because they cannot disentangle true recovery from just compensation [45, 46]. In this regard, inherent components of VRS (e.g., motion capture) offer unique tools to quantify motor recovery after stroke. For instance, in a study with chronic stroke, Kitago and colleagues observed no improvement in motor control features (mainly arm and wrist trajectory analysis and errors captured by a robotic system) after intensive constraint-induced movement therapy despite standard clinical outcomes (Action Research Arm Test and Fugl-Meyer Assessment) reported significant improvements [47]. The accumulating evidence over the last decade has had its echo in the recently formed Stroke Recovery and Rehabilitation Roundtable, which is working on defining standards for stroke recovery and rehabilitation research. This panel of experts recommends that “recovery trials need to consider serially applied kinematic/kinetic measurements alongside clinical assessments to distinguish between restitution and compensation. A core set of kinetics and kinematic outcomes needs to be established” [45].

### Videogames for neurorehabilitation

A field that is frequently confused or mixed up with virtual reality is that of videogames. A videogame is “a game played by electronically manipulating images produced by a computer program on a monitor or other display” [48]. A videogame in virtual reality, e.g. displayed in an HMD, is just “a traditional computer game – but displayed in a different medium” [12]. Indeed, the gaming industry has been the major driving force of virtual reality for its popularization.

The main advantage of videogames is their inherent capacity to motivate and enhance learning [49]. Videogames can foster learning at multiple levels (cognitive, navigation, concentration, physical) in both clinical and non-clinical populations [50]. When adapted for healthcare purposes, videogames – often called serious (video-) games – can help improve motor and cognitive conditions in different clinical populations [51, 52]. Particularly in motor rehabilitation, game-based interventions usually rely on exergames, i.e. games that involve physical exercise and that integrate motion-tracking technology (e.g. using Nintendo Wii Remotes or Microsoft Kinect) that enables interaction with the game and real-time feedback of user’s performance. It is worth nothing that not all VRS qualify for this purpose, even though they may be eligible to create interactive VRE. In particular, and depending on the targeted measure and tracked movement, low-cost motion tracking systems may not be accurate and/or reliable enough to serve as clinical outcome (see detailed reports in [53, 54]).

Interestingly, playing videogames can make structural changes in the brain, including gray matter increase and hippocampal formation, which could be used to counteract known risk factors in neurological disorders [55]. In addition, action videogames have been shown to be beneficial for a range of mental skills (including attention, faster processing of information, task switching and mental rotation) in healthy participants [56], and to improve visual acuity in the amblyopic eye [57].

### The relationship between VRE, VRS and videogames

VREs often incorporate gamification elements (e.g., rewards), to mainly increase participant’s motivation, which represents an important element in training programs for (re-)learning tasks after brain damage [58]. And vice versa, more and more innovative videogames integrate VRS initially developed for VREs, typically HMDs, motion tracking technology or, more recently, physiological information (as in so-called neurogames, which use brain or muscle signals to control basic game features [59, 60]). However, VRE-intrinsic illusions like plausibility and virtual embodiment are usually lacking in standard videogames. In the particular case of neurorehabilitation, exergames provided through standard off-the-shelf console games, where subjects can for instance use their own movements and gestures to control the movements of a virtual character, does not necessarily deliver a VRE.

The fact of using VRS elements (e.g., motion tracking or HMD) for gaming may entail the belief that those games are virtual reality applications de facto. The key point is, however, whether the use of those VRS deliver a VRE in the terms defined above. And vice versa, an application that does not use motion tracking or HMD may still deliver a strong VRE provided that some of the illusions listed above take place. In other words, the fact of using an HMD, or other peripheral such as haptic devices, does not make the *Pong* or a soccer videogame a VRE. Similarly, 360° videos projected in an HMD may generate strong feelings of presence. However, if they do not provide active interaction mechanisms, their contribution to generate strong plausibility illusion and sensorimotor contingencies would be rather limited to the moments where the soccer ball comes out of the screen towards the position of the observer (the user). This illusion will break as soon as they try to hit it. In other words, in the absence of natural interaction elements, the user may mentally feel “there” (in the soccer court) but only as long as they do not intend to move. Another category is 3D animated films such as *Avatar*. Even if we could imagine an immersive experience by exploring the computer-generated imaginary world using HMD and head tracking, it would not necessarily deliver a VRE per se, unless spectators can interact with it.

Adding VRS can help elevate a videogame or film experience to the category of VRE, e.g. making the soccer videogame life-sized with the user being placed *inside* the game and becoming (and embodying) one of the players (or one of the *Na'vi* personages in the example of the *Avatar* film), i.e. actively participating in the virtual world exploiting the sensorimotor contingencies. Then, the conceptual boundaries between videogames, films and VREs may disappear towards highly immersive, gamified and embodied VREs. Within that context, VREs should be considered as a higher-level entity since VREs can integrate games but not vice versa (games can make use of VRS though).

## Conclusion

In summary, in this short paper I have reviewed similarities, differences and synergies of VREs (virtual reality experiences), VRS (virtual reality systems) and videogames, with emphasis on neurorehabilitation. Despite the increasing link between VRS and videogames, distinction between VREs and videogames should be made when categorizing the kind of intervention. This is important for databases searches when looking for specific studies or building metareviews. Hence, I recommend a purposeful use of the term Virtual Reality Experience when the experience involves illusions, specially plausibility and virtual embodiment, irrespectively whether the experience is gamified or not. More importantly, when reporting on the effectiveness of VREs and/or videogames for neurorehabilitation, it is highly recommended describing and evaluating the specific features encompassing the intervention (e.g. role of mirror visual feedback, movement augmentation, motor-auditory coupling, virtual embodiment, etc.) rather than evaluating virtual reality or videogames as a whole. Finally, VRS fall under a different category than VREs and videogames: while the latter refer to the generated experience and content, VRS refer to the technology (mainly hardware) used to create those experiences and content. This disambiguation should help reduce confusion in a field where large univariable and well-controlled clinical trials are scarce.
